# From mild behavioral impairment-checklist (MBI-C) to MBI-distress (MBI-D): a paired assessment and clinical correlates of domain-specific caregiver distress in MCI due to AD

**DOI:** 10.3389/frdem.2026.1736570

**Published:** 2026-02-24

**Authors:** Efthalia Angelopoulou, Niki Tsinia, Maria Hatzopoulou, Akylina Despoti, Vasiliki Kamtsadeli, Marina Papadogiani, Evangelia Stanitsa, Vasilis Kyriakidis, Sokratis Papageorgiou, John D. Papatriantafyllou

**Affiliations:** 1 1st Department of Neurology, Aiginition University Hospital, Medical School, National and Kapodistrian University of Athens, Athens, Greece; 2 Third Age Day Care Center IASIS, Athens, Greece; 3 1st Department of Psychiatry, Aiginition University Hospital, Medical School, National and Kapodistrian University of Athens, Athens, Greece

**Keywords:** Alzheimer’s disease, caregiver burden, dementia, mild behavioral impairment, mild behavioral impairment-checklist, mild cognitive impairment

## Abstract

**Background:**

Mild behavioral impairment (MBI) captures later-life onset neuropsychiatric symptoms (NPS) that may herald neurodegeneration. The emotional impact of these early behavioral changes on caregivers is under-measured in pre-dementia care.

**Objective:**

To develop a brief, domain-aligned caregiver distress scale for MBI (MBI-D) and examine clinical correlates of MBI-related caregiver distress in mild cognitive impairment due to AD (MCI-AD).

**Methods:**

One hundred and four participant-informant dyads with MCI-AD at a Greek memory clinic were included. Caregivers completed the Greek MBI-C and the new five-item MBI-D (one item per ISTAART-AA MBI domain). Internal consistency (Cronbach’s *α*), non-parametric tests, and Spearman correlations assessed bivariate associations. Multiple linear regression identified independent correlates of MBI-D total. Prespecified covariates were age, education, sex, global cognition (MMSE or ACE-R), disease duration, and MBI-C (total or domains).

**Results:**

Internal consistency of the MBI-D was moderate (*α* = 0.617; standardized *α* = 0.627; mean inter-item *r* = 0.25). MBI-D total correlated strongly with MBI-C total (*ρ* = 0.789, *p* < 0.001), and each MBI-D domain correlated with its corresponding MBI-C domain (*ρ* = 0.478–0.850, all *p* < 0.001). Disease duration was associated with MBI-D total and with apathy-related distress (*ρ* = 0.302, *p* = 0.002 and *ρ* = 0.392, *p* < 0.001, respectively). In multivariable regression, MBI-C total and education were independent predictors of MBI caregiver distress (*β* = 0.804, *p* < 0.001, and *β* = 0.135, *p* = 0.017, respectively). In the MBI-C domains model, impulse dyscontrol, apathy and emotional dysregulation independently related to higher distress (*B* = 0.513, *β* = 0.482, *p* < 0.001, *B* = 0.315, *β* = 0.278, *p* < 0.001, and *B* = 0.289, *β* = 0.227, *p* = 0.001 respectively), while cognitive performance (MMSE and ACE-R) did not have a significant impact.

**Conclusion:**

The MBI-D, strongly coupled with MBI-C, is a concise, clinically practical and scalable measure of MBI-related caregiver distress in MCI-AD, capturing both symptom burden and domain-specific distress in a single administration. Impulsivity, apathy, and affective dysregulation are highlighted as priority targets for early, caregiver-focused interventions advancing innovative, prevention-oriented dementia care delivery.

## 1 Introduction

In dementia, neuropsychiatric symptoms (NPS) such as agitation, apathy, disinhibition and hallucinations have been consistently linked to increased morbidity, mortality, institutionalization, and caregiver burden (Steinberg et al., 2008). Among the various physical, societal and economic dimensions of this burden, the caregivers’ subjective feelings and coping capacity play a central role in their perceived overload. The sustained demands of caregiving may lead to reduced quality of life, social withdrawal, family conflicts, anxiety and depression (Huang, 2022). These emotional responses can in turn exacerbate or trigger the behavioral symptoms in people with dementia (Zhang et al., 2022). NPS often present unpredictably and fluctuate throughout the disease course, making them challenging to manage. They are frequently associated with adverse outcomes such as injuries and falls, further contributing to caregiver distress (Basu and Mukhopadhyay, 2022). While several instruments exist to assess caregiver burden, such as the Zarit Burden Interview (ZBI), the Caregiver Burden Index (CBI), the Burden Scale for Family Caregivers (BSFC), and the Dementia Caregiver Burden Scale (DCBS) (Tu et al., 2022), these tools mainly target caregivers of people with dementia, and they do not specifically measure NPS-related distress.

The Neuropsychiatric Inventory (NPI) is the most widely used tool for assessing NPS in patients with dementia both in clinical practice and research. It is an interview-based instrument that evaluates the frequency and severity of 10 to 12 NPS domains, as reported by a caregiver over the preceding 4 weeks (Saari et al., 2022). Complementing the NPI, the NPI Distress Scale (NPI-D) was designed to quantitatively evaluate the emotional or psychological distress of the caregivers in response to each NPS domain, in a 6-point Likert scale (Kaufer et al., 1998). Both the NPI and NPI-D have been validated in multiple languages, offering a practical tool to capture NPS and their impact on caregivers primarily in patients with dementia (Saari et al., 2022).

While NPS have traditionally been associated with later stages of Alzheimer’s disease (AD), increasing attention is directed toward prodromal and preclinical phases. In this context, many individuals may exhibit subtle behavioral changes prior to the onset of cognitive impairment. A study by Lyketsos et al. (2002) reported that about one in two patients with MCI display at least one NPS at the initial presentation of cognitive symptomatology. According to the 2018 (Jack et al., 2018) and the updated 2024 (Jack et al., 2024) National Institute on Aging and Alzheimer’s Association (NIA-AA) Research Framework criteria, Stage 2 AD may be characterized primarily by mild behavioral alterations rather than clear cognitive deficits. Notably, while the annual conversion rate from mild cognitive impairment (MCI) to dementia is approximately 10–15%, co-occurring NPS may elevate this risk to 25% (Rosenberg et al., 2013). These findings highlight the importance of early identification and monitoring of NPS in older adults, as they may represent an at-risk state and/or early indicators of a neurodegenerative disease (Rosenberg et al., 2013).

According to the International Society to Advance Alzheimer’s Research and Treatment-AA (ISTAART-AA), mild behavioral impairment (MBI) is defined as the later-life emergence of NPS in individuals aged 50 years or older, representing a clear change from their usual behavior or personality, and persisting for at least 6 months (Ismail et al., 2017). These NPS are categorized into five domains: (i) decreased motivation (apathy, aspontaneity, indifference), (ii) emotional dysregulation (dysphoria, anxiety, changeability, euphoria), (iii) impulse dyscontrol (agitation, obsessiveness, stimulus bind behavior, gambling, behavioral perseveration), (iv) social inappropriateness (loss of empathy and insight, rigidity), and (v) abnormal perception or thought content (delusions, hallucinations). These behavioral alterations should not be explained by a preexisting psychiatric disorder, medical condition, medication side effects or substance use. The individual should remain functionally independent, with either normal cognition or MCI (Ismail et al., 2017). The MBI Checklist (MBI-C) is a 34-item validated tool specifically designed to operationalize the ISTAART-AA criteria, offering a domain-based approach to identifying MBI that can be completed by caregivers, clinicians or the individual themselves.

Despite the increasing recognition of MBI, its emotional impact on caregivers remains poorly understood, and there is currently no specific scale to assess MBI-related caregiver distress. While the NPI-D is a well-established tool in dementia, its utility in populations without dementia is limited, as the NPI was not originally designed for use in functionally independent, cognitively intact individuals. Conversely, while the MBI-C effectively captures early behavioral symptoms in such populations, it does not assess the associated emotional burden on caregivers.

The expression, severity and consequences of NPS vary across the stages of neurodegeneration (Chen et al., 2021). Depression, anxiety, irritability and apathy are frequently observed in prodromal and mild stages of AD, whereas delusions, hallucinations, euphoria and aberrant motor behavior such as wandering tend to appear in more advanced disease stages (Chen et al., 2021). In earlier phases, caregivers of individuals with MBI may encounter unique challenges. The behavioral changes may be subtle, misattributed to personality, or dismissed entirely, yet still result in substantial emotional distress and strain on the caregiving relationship (Mank et al., 2023). Moreover, caregivers of individuals with MBI may be less likely to receive formal recognition or support, potentially leaving their psychological needs unmet.

The development of a dedicated MBI-related caregiver distress scale could facilitate earlier psychosocial and clinical interventions and ultimately improve outcomes for both caregivers and patients. Quantifying the burden associated with MBI and identifying its contributing factors would also support efforts to detect caregivers at higher risk for mental health concerns and burnout. Such evidence could inform public health strategies and guide the development of targeted support services. Therefore, a concise, reliable and MBI-specific distress scale that is suitable for both clinical and research settings is needed.

This study aimed to introduce and evaluate the psychometric properties of a new scale (MBI-D) that integrates the MBI-C, the standard tool for assessing MBI based on ISTAART-AA criteria, with the NPI-D, which quantifies caregiver distress related to specific NPS in neurodegenerative diseases. By combining these two validated instruments, the MBI-D captures both symptomatology and caregiver distress in a unified framework, facilitating time-efficient and clinically relevant assessment in pre-dementia populations. Furthermore, we aimed to characterize the distribution of caregiver distress across the five MBI domains and identify clinical correlates of MBI-D total using multivariable models that included age, education, sex, global cognition, disease duration, and either MBI-C total or the five domain scores.

## 2 Methods

### 2.1 Study participants

This cross-sectional study is a substudy of our previously published cohort, in which the recruitment protocol, measures, and procedures have been described in detail (Angelopoulou et al., 2025). For the purposes of the present analysis, we included only participant-informant dyads with MCI-AD and their caregivers.

One hundred and four participant-informant dyads with MCI-AD, aged ≥50 years, assessed at the Third Age Day Care Center IASIS between 2019–2023 were included. Each participant was accompanied by a close informant/caregiver (CG). Diagnostic classification followed the 2011 NIA-AA criteria. Senior memory specialists at the Memory Clinic established MCI-AD diagnoses based on clinical history, standardized neuropsychological profiles, and ancillary investigations.

Exclusion criteria were diagnosis of dementia, lack of a reliable informant, inability to communicate effectively in Greek, and evidence suggestive of non-AD cognitive impairment (e.g., brain MRI demonstrating significant cerebrovascular pathology compatible with vascular cognitive impairment).

All MCI-AD participants underwent a comprehensive evaluation that included a structured clinical interview, neuropsychological testing, neuroimaging (brain CT or MRI), and additional diagnostic work-up when clinically indicated. Demographic characteristics of the MCI-AD patients, including age, sex, and years of education, were also systematically recorded and used in all subsequent analyses.

The study was approved by the Scientific Committee of the Third Age Day Care Center IASIS (Protocol No. 03, Approval Date: December 20, 2019). Written informed consent was obtained from all participants prior to enrollment.

### 2.2 The Mild Behavioral Impairment Caregiver Distress (MBI-D) scale

The MBI-C is the first instrument specifically designed to assess MBI in older adults, in accordance with the ISTAART-AA criteria (Jack et al., 2024). This two-page questionnaire comprises 34 items grouped into the five core MBI domains: reduced motivation (apathy), emotional dysregulation, impulse dyscontrol, social inappropriateness, and abnormal perception or thought content (Jack et al., 2024). The apathy domain includes six items addressing cognitive, emotional, and behavioral aspects. Emotional dysregulation is assessed through six items, including four targeting depressive symptoms (anhedonia, guilt, low mood, and hopelessness), along with one item each for anxiety and panic. The impulse dyscontrol domain includes 12 items that evaluate manifestations such as agitation, irritability, impulsive behaviors, altered reward processing, and recklessness. The social inappropriateness domain contains five items focusing on empathy, tact, and social sensitivity, while the abnormal thoughts and perceptions domain consists of five items related to hallucinations and delusions, including suspiciousness and grandiosity (Jack et al., 2024).

The MBI-C can be completed by an informant, the individual themselves, or a clinician (Jack et al., 2024). At the beginning of the questionnaire, it is clearly stated that the behavior should signify a noticeable and sustained change from the person’s long-standing behavior, persisting for at least 6 months, either continuously or episodically (Jack et al., 2024). Each item requires a “no” or “yes” response with a severity rating (0 = no, 1 = mild, 2 = moderate, 3 = severe) (Jack et al., 2024). The total MBI-C score, ranging from 0 to 102, is computed by summing the severity scores across all items, while domain-specific scores are obtained by summing the scores within each domain. Higher scores indicate greater behavioral change. The MBI-C is applicable in both clinical practice and research, aiding in the early detection and monitoring of NPS in populations at risk for dementia (Jack et al., 2024). The tool has been validated in individuals with MCI (Rosenberg et al., 2013) and subjective cognitive decline (SCD) (Ismail et al., 2017), with suggested cutoff scores of 6.5 and 8.5, respectively, to define MBI. While its applicability has been confirmed in cognitively normal populations, cutoff values in these cases, as well as domain-specific cutoff values have yet to be established (Chen et al., 2021). In the present study, we applied an MBI-C cutoff score of 9.5, based on our previous findings in the same Greek cohort, where this threshold demonstrated optimal diagnostic accuracy for distinguishing individuals with MCI-AD from cognitively normal participants (Angelopoulou et al., 2025). Given that MBI expression and optimal cutoff values may vary according to clinical population and sociocultural context, the use of a cohort-specific cutoff was considered methodologically appropriate. The MBI-C is publicly available at https://mbitest.org/, and it has been translated and validated in several languages.

The NPI is a validated, interview-based tool developed to evaluate the frequency and severity of common NPS in patients with dementia over the preceding 4 weeks, based on caregiver reports (Saari et al., 2022). The original NPI version includes 10 NPS domains: delusions, hallucinations, dysphoria/depression, agitation/aggression, anxiety, apathy/indifference, euphoria/elation, irritability/lability, disinhibition, and aberrant motor behavior, with two additional items for sleep and appetite disturbances introduced later. For each NPI domain, a screening question is posed with a binary “yes” or “no” response. If the response is positive, six to nine subquestions further explore symptom characteristics. Each domain is rated for frequency (1 = occasionally, 2 = often, 3 = frequently, 4 = very frequently) and severity (1 = mild, 2 = moderate, 3 = severe), yielding a composite domain score (frequency × severity) ranging from 1 to 12. The total NPI score is the sum of all positive domain scores, with a maximum of 120 for the 10-domain version and 144 for the 12-domain version (Saari et al., 2022).

The NPI-D is a validated supplementary scale that measures caregiver emotional distress associated with each NPS domain captured by NPI for patients with dementia (Kaufer et al., 1998). After evaluating the frequency and severity of NPS, caregivers are asked to rate the psychological or emotional burden associated with each symptom on a 6-point scale: 0 (not distressing), 1 (minimally distressing), 2 (mildly distressing), 3 (moderately distressing), 4 (very severely or severely distressing), and 5 (extremely distressing) (Kaufer et al., 1998).

The newly developed MBI-D aimed to measure the psychological or emotional distress associated with each of the five MBI domains, as defined by the ISTAART-AA criteria and assessed by the MBI-C: decreased motivation, emotional dysregulation, impulse dyscontrol, social inappropriateness, and abnormal perception or thought content. The quantification of MBI-related distress was based on the NPI-D construct. Two bilingual dementia experts familiar with MBI (JP, psychiatrist, MH, clinical neuropsychologist) developed the MBI-D items in Greek, based on the terminology used in the Greek versions of the MBI-C and NPI (Angelopoulou et al., 2025; Politis et al., 2004).

To examine whether the MBI-D accurately captures the intended construct (caregiver emotional or psychological distress related to MBI symptoms) and evaluate its content validity, a review was conducted by an expert panel comprising a neuropsychologist (AD) and a behavioral neurologist (SP) via joint discussion.

Following confirmation of any symptoms present in each domain using the MBI-C, CG were asked to rate the extent of emotional distress these symptoms had caused them over the past 6 months. Drawing from the structure of the NPI-D, five corresponding items were developed and placed directly after the domain-specific MBI-C items. These five items were phrased in English as follows: “If present, how emotionally distressing have the symptoms of reduced motivation been for you?,” “If present, how emotionally distressing have the symptoms of emotional changes been for you?,” “If present, how emotionally distressing have the impulsive behavioral symptoms been for you?,” “If present, how emotionally distressing have the symptoms of socially inappropriateness been for you?,” and “If present, how emotionally distressing have the symptoms of abnormal thoughts or perceptions been for you?”

Distress is rated on a 6-point Likert scale (0 = no distress, 1 = minimally distressing, 2 = mildly distressing, 3 = moderately distressing, 4 = severely distressing, 5 = very severely or extremely distressing), mirroring the NPI-D format. The total MBI-D score is calculated by summing the scores across the five domains, ranging from 0 to 25, with higher scores indicating greater caregiver distress.

Reliability was examined through internal consistency analysis, assessing the degree of correlation among items as a unified scale.

### 2.3 Cognitive and behavioral assessment

The neuropsychological assessment included the Mini-Mental State Examination (MMSE), the most widely used screening tool for global cognitive assessment, and the more detailed Addenbrooke’s Cognitive Examination-Revised (ACE-R). MMSE is a 30-item instrument evaluating orientation, attention, memory (immediate/delayed recall), language, calculation, and visuoconstructional skills, with scores ranging 0–30 (lower scores indicating greater impairment). ACE-R assesses five domains: attention/orientation, memory, verbal fluency, language, and visuospatial function, yielding a 100-point composite score (higher scores denoting better performance). To assess NPS and related caregiver distress in the context of MBI, the CG of each participant completed the MBI-C and MBI-D, respectively.

### 2.4 Statistical analysis

Demographic and clinical characteristics were summarized using descriptive statistics, with frequencies reported for categorical variables. Distributional assumptions were assessed using Shapiro–Wilk tests and inspection of histograms and Q–Q plots. Depending on the distribution, means and standard deviations or medians and interquartile ranges were calculated for continuous variables. Because MBI-D scores were predominantly non-normal, primary inferential analyses used non-parametric tests. Patients’ sex differences in MBI-D total and domain scores were tested with the Mann–Whitney *U* test (two-tailed). Associations of MBI-D (total and domain scores) with patients’ age, education (years), MMSE, ACE-R, and disease duration (months) were examined using Spearman’s *ρ*. Correlation coefficients (Spearman’s *ρ*) are reported as measures of effect size for all bivariate associations.

We also computed Spearman correlations between MBI-C total and MBI-D total, and between each MBI-D domain and its matching MBI-C domain (e.g., emotional dysregulation with emotional dysregulation). In supplementary analyses, correlations between MBI-D domain scores and MBI-C total score were also performed. Cross-domain pairs (e.g., MBI-D emotional dysregulation with MBI-C decreased motivation) were not tested.

Internal consistency of the 5-item MBI-D was evaluated with Cronbach’s *α*, as a standard psychometric index to examine whether the items function coherently when combined into a total caregiver distress score. Although the items correspond to distinct MBI domains, this analysis was performed to support the psychometric justification for computing a global MBI-D score alongside domain-specific distress ratings.

To identify independent correlates of MBI caregiver distress, we run a multiple linear regression analysis with MBI-D total score as the dependent variable and patients’ age, education, sex (1 = female), MMSE, disease duration, and MBI-C total as predictors (Enter method). Collinearity diagnostics (Tolerance/VIF) and residual checks were performed.

The statistical significance level was set at *p* ≤ 0.05. All analyses were performed with IBM SPSS Statistics 28.0.

## 3 Results

### 3.1 Demographics and clinical characteristics of MCI-AD patients

One hundred and four participant-informant dyads with MCI-AD were included. Table 1 summarizes patients’ demographics and clinical characteristics: age (years), education (years), disease duration (months from symptom onset to assessment), sex (male/female), global cognition (MMSE, ACE-R), MBI-C total and domain scores, and MBI frequency using the 9.5 cutoff.

**Table 1:** Descriptive characteristics of MCI-AD patients [demographics, cognitive performance (MMSE/ACE-R), and MBI-C total and domain scores].

Characteristic	Patients with MCI-AD (*n* = 104)
Age (years), Mdn ± IQR	77 ± 9
Disease duration (months), M ± SD	30.12 ± 19.10
Sex (male/female %)	39.4/60.6
Education (years), Mdn ± IQR	10.44 ± 4.05
MMSE, Mdn ± IQR	26 ± 2
ACE-R, M ± SD	72.77 ± 8.93
MBI-C total score, Mdn ± IQR	12.5 ± 12
MBI-C emotional dysregulation domain score, Mdn ± IQR	4.5 ± 5
MBI-C decreased motivation domain score, Mdn ± IQR	2 ± 5
MBI-C impulse dyscontrol domain score, Mdn ± IQR	4 ± 5
MBI-C social inappropriateness domain score, Mdn ± IQR	0 ± 2
MBI-C abnormal perception or thought content domain score, Mdn ± IQR	0 ± 1
MBI (yes/no %)^a^	70/34

a Using the MBI-C total score cutoff of 9.5; M, mean; SD, standard deviation; Mdn, median; IQR, interquartile range; MCI-AD, mild cognitive impairment due to Alzheimer’s disease; MMSE, Mini-Mental State Examination; ACE-R, Addenbrook’s Cognitive Examination-Revised; MBI-C, mild behavioral impairment checklist.

### 3.2 The final version of the Greek MBI-D

The developed Greek MBI-D version, which accompanies MBI-C, and a translated English version are depicted in Suppelementary Tables A1, A2.

### 3.3 Psychometric properties of the Greek MBI-D

Regarding content validity, the expert review confirmed the scale’s conceptual clarity, appropriateness and relevance. The 5-item MBI-D showed a Cronbach’s alpha of 0.617, indicating moderate internal consistency (listwise deletion, no missing cases). Across the five MBI-D items, the mean inter-item correlation was 0.25 (10 pairs; range −0.10 to 0.50). The strongest pairwise associations were between emotional dysregulation and decreased motivation (*r* = 0.50) and between decreased motivation and impulse dyscontrol (*r* = 0.40). Correlations involving social inappropriateness were modest (e.g., *r* = 0.26–0.39), while impulse dyscontrol with abnormal perceptions/thought content was near zero (*r* = −0.10).

### 3.4 MBI caregiver distress and patients’ demographic and clinical characteristics

Median MBI-D total was 8 (IQR 5–11.75, Table 2). Domain medians were: emotional dysregulation 3 (2–4), decreased motivation 2 (0–3), impulse dyscontrol 2 (0–3), social inappropriateness 0 (0–1), and abnormal perception/thought content 0 (0–3). None of the MBI-D domain scores differed by patients’ sex (Mann–Whitney *U*; all *p* > 0.05). Patients’ age was not correlated with MBI-D total or domains scores (Spearman *ρ* = 0.011–0.127, all *p* > 0.05). Patients’ education was positively associated only with the decreased motivation domain (*ρ* = 0.263, *p* = 0.007); no associations were observed for the other MBI-D domain scores (all *p* > 0.05) (see Table 2).

**Table 2:** Medians (IQR) of MBI-D total and domain scores and their associations with MCI-AD patients’ sex, age, and education.

Characteristic	All (*n* = 104)	Patients’ sex^a^			Patients’ age (years)^b^ (rho, *p*-value)	Patients’ education level (years)^b^ (rho, *p*-value)
		Males (*n* = 41)	Females (*n* = 63)	*p*-value		
MBI-D total score	8 (5, 11,75)	9 (6, 12)	7 (5, 11)	0.105	0.063 (0.525)	0.156 (0.114)
MBI-D emotional dysregulation domain score	3 (2, 4)	3 (2, 5)	3 (2, 3)	0.088	0.127 (0.199)	0.032 (0.744)
MBI-D decreased motivation domain score	2 (0, 3)	2 (0, 4)	2 (0, 3)	0.279	−0.093 (0.345)	**0**.**263 (0**.**007)**
MBI-D impulse dyscontrol domain score	2 (0, 3)	2 (0, 4)	2 (0, 3)	0.370	0.071 (0.476)	0.090 (0.365)
MBI-D social inappropriateness domain score	0 (0, 1)	0 (0, 2)	0 (0, 1)	0.207	0.100 (0.311)	0.031 (0.757)
MBI-D abnormal perception or thought content domain score	0 (0, 3)	0 (0, 2)	0 (0, 3)	0.892	0.011 (0.912)	0.057 (0.564)

a Mann–Whitney *U*.

b Spearman.Bold values are indicating statistically significant results.

MBI-D total and domain scores showed no associations with cognitive performance (MMSE or ACE-R, all *p* > 0.05, Table 3). In contrast, disease duration positively correlated with MBI-D total score (*ρ* = 0.302, *p* = 0.002) and with the MBI-D score in the decreased motivation domain (*ρ* = 0.392, *p* < 0.001). The association with MBI-D score in social inappropriateness trended toward significance (*ρ* = 0.190, *p* = 0.053), while the remaining domains were not significantly correlated with duration (all *p* > 0.139).

**Table 3:** Spearman correlations between MBI-D caregiver distress (total and domain scores) and MMSE, ACE-R, and disease duration of MCI-AD patients.

Characteristic	MMSE (rho, *p*-value)	ACE-R (rho, *p*-value)	Disease duration (months) (rho, *p*-value)
MBI-D total score	0.038 (0.702)	0.046 (0.645)	**0.302 (0.002)**
MBI-D emotional dysregulation domain score	0.054 (0.586)	0.107 (0.280)	0.146 (0.139)
MBI-D decreased motivation domain score	0.037 (0.710)	−0.059 (0.552)	**0.392 (<0.001)**
MBI-D impulse dyscontrol domain score	−0.046 (0.642)	0.007 (0.940)	0.098 (0.324)
MBI-D social inappropriateness domain score	−0.057 (0.567)	0.035 (0.726)	0.190 (0.053)
MBI-D abnormal perception or thought content domain score	0.114 (0.249)	0.035 (0.727)	0.087 (0.379)

Bold values are indicating statistically significant results.

### 3.5 ΜΒΙ caregiver distress and patients’ MBI symptoms

Spearman analyses showed a strong association between MBI-D total and MBI-C total scores (*ρ* = 0.789, *p* < 0.001, Table 4). Domain-level MBI-D scores correlated positively with their corresponding MBI-C domains: emotional dysregulation (*ρ* = 0.524, *p* < 0.001), decreased motivation (*ρ* = 0.850, *p* < 0.001), impulse dyscontrol (*ρ* = 0.640, *p* < 0.001), social inappropriateness (*ρ* = 0.720, *p* < 0.001), and abnormal perception/thought (*ρ* = 0.478, *p* < 0.001). All correlations between MBI-D domain scores and their corresponding MBI-C domain scores remained statistically significant even after Bonferroni correction.

**Table 4:** Spearman correlations between MBI-D caregiver distress (total and domain scores) and MBI-C symptom severity (total and corresponding domains).

Characteristic	MBI-C total score (rho, *p*-value)	MBI-C emotional dysregulation domain score (rho, *p*-value)	MBI-C decreased motivation domain score (rho, *p*-value)	MBI-C impulse dyscontrol domain score (rho, *p*-value)	MBI-C social inappropriateness domain score (rho, *p*-value)	MBI-C abnormal perception or thought content domain score (rho, *p*-value)
MBI-D total score	**0.789 (<0.001)**	—	—	—	—	—
MBI-D emotional dysregulation domain score	0.696 (<0.001)	**0.524 (<0.001)**	—	—	—	—
MBI-D decreased motivation domain score	0.688 (<0.001)	—	**0.850 (<0.001)**	—	—	—
MBI-D impulse dyscontrol domain score	0.534 (<0.001)	—	—	**0.640 (<0.001)**	—	—
MBI-D social inappropriateness domain score	0.492 (<0.001)	—	—	—	**0.720 (<0.001)**	—
MBI-D abnormal perception or thought content domain score	0.352 (<0.001)	—	—	—	—	**0.478 (<0.001)**

Bold values are indicating statistically significant results.

Supplementary Spearman’s correlation analyses demonstrated significant associations between MBI-C total score and all MBI-D domain scores (*ρ* = 0.35–0.70), all of which remained significant after Bonferroni correction.

### 3.6 Regression analyses

A multiple linear regression was run to identify correlates of caregiver distress (MBI-D total) with patients’ age, education, sex (1 = female), MMSE, disease duration, and MBI-C total entered simultaneously (Enter method, Table 5). The model was significant and accounted for 70.3% of the variance in MBI-D [adjusted *R*^2^ = 0.684; *F*(6,97) = 38.188, *p* < 0.001]. MBI-C total emerged as the dominant independent correlate (*β* = 0.804, *p* < 0.001), indicating that higher overall MBI symptom severity was strongly associated with greater caregiver distress. Education showed a smaller but statistically significant positive association (*β* = 0.135, *p* = 0.017). Age and disease duration exhibited positive trends but did not reach conventional significance (both *p* ≈ 0.08). Sex and MMSE were not independently associated with MBI-D (both *p* > 0.50). Collinearity diagnostics were acceptable (VIF = 1.02–1.11; Tolerance = 0.897–0.983). Model residuals were approximately centered with no gross heteroscedasticity on visual inspection, and the standard error of the estimate was 2.910. Overall, adding disease duration to the model did not materially alter the strong association between MBI-C total and caregiver distress.

**Table 5:** Multiple linear regression predicting MBI-D caregiver distress (total score) from patients’ age, education, sex, MMSE, disease duration, and MBI-C total score.

Predictor	*B*	SE	*β*	95% CI for *B*	*t*	*p*-value	VIF
Patients’ age (years)	0.069	0.038	0.100	−0.007, 0.145	1.789	0.077	1.020
Patients’ education (years)	0.173	0.071	0.135	0.031, 0.315	2.421	**0.017**	1.017
Patients’ sex (1 = female, 0 = male)	0.373	0.616	0.035	−0.849, 1.595	0.606	0.546	1.112
MMSE	−0.083	0.186	−0.25	−0.452, 0.287	−0.444	0.658	1.052
Disease duration (months)	0.028	0.016	0.103	−0.003, 0.059	1.794	0.076	1.079
MBI-C total score	0.341	0.025	0.804	0.292, 0.390	13.761	**<0.001**	1.114

Model fit: *R* = 0.838, *R*^2^ = 0.703, Adjusted *R*^2^ = 0.684, SE of estimate = 2.910; *F*(6, 97) = 38.188, *p* < 0.001; Constant: *B* = −2.793, SE = 6.067, *p* = 0.646; Collinearity: Tolerance 0.897–0.983; VIF 1.02–1.11. Bold values are indicating statistically significant results.

**Figure 1:**
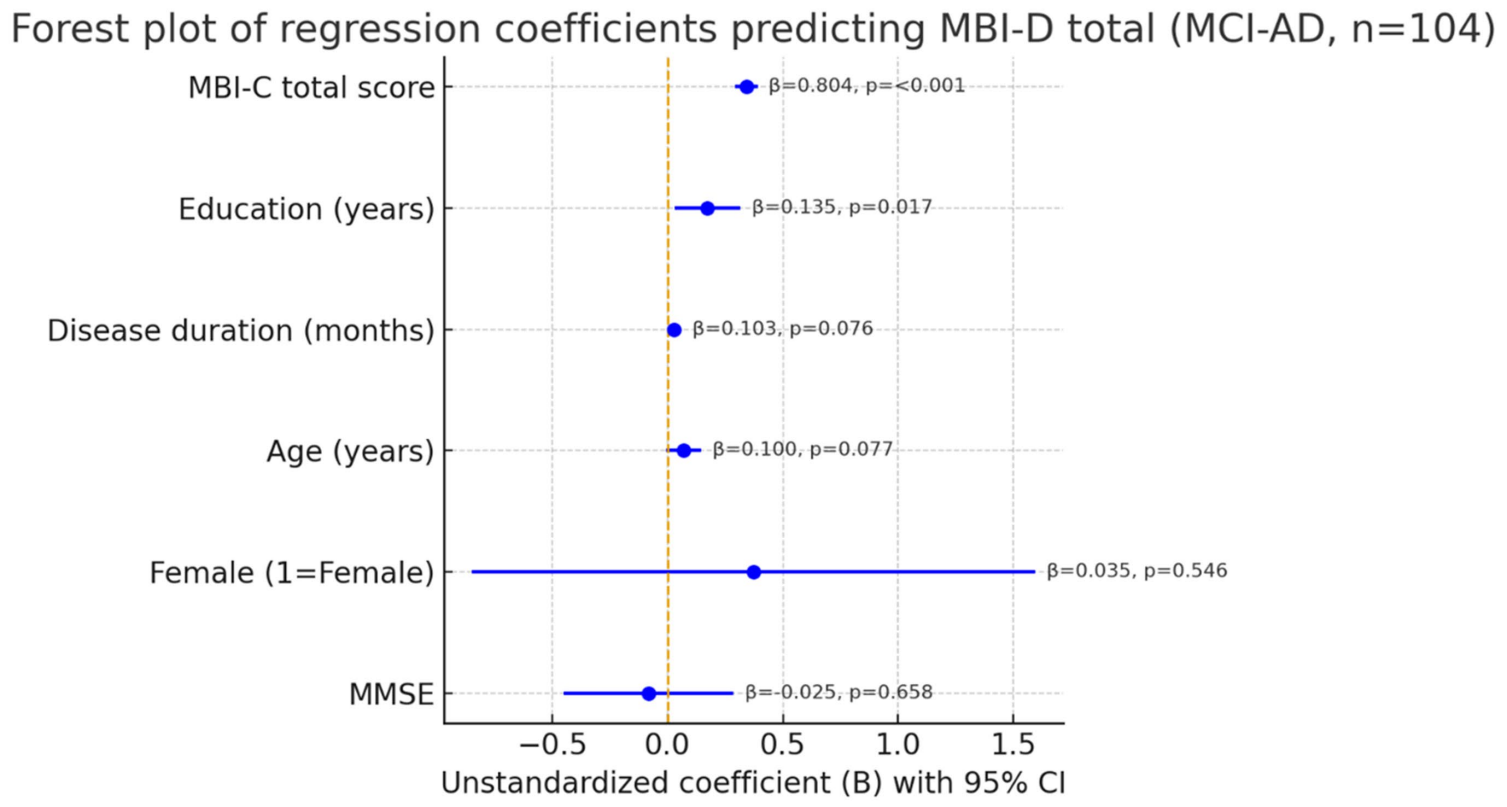
Forest plot of unstandardized coefficients (*B*) with 95% confidence intervals from the multiple linear regression predicting MBI-D total in MCI-AD. Predictors: patients’ age, education, sex (1 = female), MMSE, disease duration, MBI-C total. Model fit: *R*^2^ = 0.703 (adjusted *R*^2^ = 0.684), *F*(6,97) = 38.188, *p* < 0.001.

In a supplementary analysis including ACE-R instead of MMSE, the multiple linear regression model was significant and explained 70.7% of the variance in MBI-D [adjusted *R*^2^ = 0.689; *F*(6,97) = 39.041, *p* < 0.001; SE of estimate = 2.887]. MBI-C total remained the dominant independent correlate (*β* = 0.812, *p* < 0.001). Education showed a smaller but significant positive association (*β* = 0.130, *p* = 0.021), with age being positively associated with distress (*β* = 0.115, *p* = 0.042). ACE-R, patients’ sex, and disease duration were not independently associated (all *p* ≥ 0.10). Collinearity diagnostics were acceptable (Tolerance ≈0.88–0.99; VIF ≈1.01–1.13) (see Figure 1).

**Figure 2:**
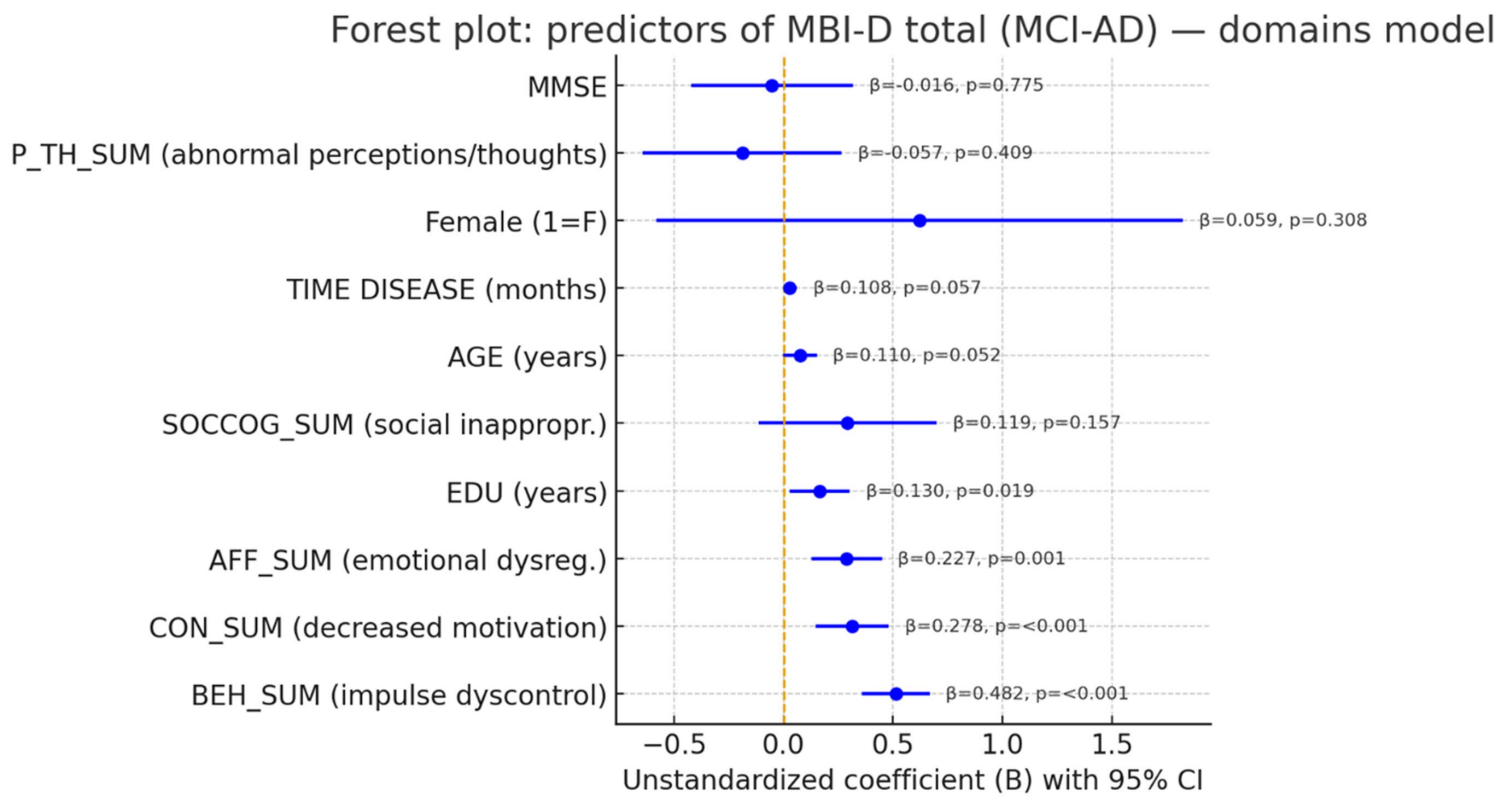
Forest plot of unstandardized coefficients (*B*) with 95% confidence intervals from the multiple linear regression predicting MBI-D total. Predictors: patients’ age, education, sex (1 = female), MMSE, disease duration, and MBI-C domain scores (AFF_SUM, CON_SUM, BEH_SUM, SOCCOG_SUM, P_TH_SUM). Model fit: adjusted *R*^2^ = 0.706; *F*(10,93) = 25.684; *p* < 0.001.

We run another multiple linear regression analysis model, including all MBI-C domain scores instead of MBI-C total score (Table 6 and Figure 2). The model was significant and explained 73.4% of the variance in caregiver distress [adjusted *R*^2^ = 0.706; *F*(10,93) = 25.684, *p* < 0.001; SE of estimate = 2.809]. Among MBI-C domains, impulse dyscontrol (*B* = 0.513, 95% CI 0.357–0.668, *β* = 0.482, *p* < 0.001), decreased motivation (*B* = 0.315, 0.147–0.482, *β* = 0.278, *p* < 0.001), and emotional dysregulation (*B* = 0.289, 0.129–0.449, *β* = 0.227, *p* = 0.001) were independent correlates of higher MBI-D. Social inappropriateness (*B* = 0.292, −0.114–0.698, *β* = 0.119, *p* = 0.157) and abnormal perception/thought (*B* = −0.189, −0.643–0.264, *β* = −0.057, *p* = 0.409) were not significant. Of the covariates, education showed a positive association (*B* = 0.166, 0.029–0.304, *β* = 0.130, *p* = 0.019); patients’ age and disease duration trended positively but did not reach conventional significance (age: *p* = 0.052; duration: *p* = 0.057). Sex and MMSE were not associated (both *p* ≥ 0.308). Collinearity diagnostics indicated no severe multicollinearity (Tolerance ≥0.410; VIF ≤2.44), and residuals were approximately centered.

**Table 6:** Multiple linear regression predicting MBI-D caregiver distress (total score) from patients’ age, education, sex, MMSE, disease duration, and MBI-C domain scores.

Predictor	*B*	SE	*β*	95% CI for *B*	*t*	*p*-value	VIF
Patients’ age (years)	0.075	0.038	0.110	−0.001, 0.152	1.969	0.052	1.095
Patients’ education (years)	0.166	0.069	0.130	0.029, 0.304	2.398	**0.019**	1.030
Patients’ sex (1 = female, 0 = male)	0.622	0.606	0.059	−0.582, 1.826	1.026	0.308	1.157
MMSE	−0.053	0.186	−0.016	−0.423, 0.316	−0.287	0.775	1.128
Disease duration (months)	0.029	0.015	0.108	−0.001, 0.060	1.930	0.057	1.102
MBI-C emotional dysregulation score	0.289	0.081	0.227	0.129, 0.449	3.585	**0.001**	1.408
MBI-C decreased motivation score	0.315	0.085	0.278	0.147, 0.482	3.720	**<0.001**	1.949
MBI-C impulse dyscontrol score	0.513	0.078	0.482	0.357, 0.668	6.547	**<0.001**	1.894
MBI-C social inappropriateness score	0.292	0.205	0.119	−0.114, 0.698	1.428	0.157	2.437
MBI-C abnormal perception or thought content score	−0.189	0.228	−0.057	−0.643, 0.264	−0.830	0.409	1.624

Model fit: *R* = 0.857; *R*^2^ = 0.734; Adjusted *R*^2^ = 0.706; SE of estimate = 2.809; *F*(10, 93) = 25.684, *p* < 0.001. Constant: *B* = −4.303, SE = 6.132, *p* = 0.485. Collinearity: Tolerance ≥0.410; VIF ≤2.44. Bold values are indicating statistically significant results.

In a supplementary analysis, including ACE-R instead of MMSE, caregiver distress (MBI-D total) remained independently associated with impulse dyscontrol (*B* = 0.505, 95% CI 0.352–0.658, *β* = 0.475, *p* < 0.001), decreased motivation (*B* = 0.322, 0.156–0.489, *β* = 0.285, *p* < 0.001), and emotional dysregulation (*B* = 0.286, 0.128–0.443, *β* = 0.225, *p* = 0.001). Among covariates, patients’ education was significant (*B* = 0.159, *β* = 0.124, *p* = 0.023) and age became marginally stronger—now significant (*B* = 0.088, 0.012–0.164, *β* = 0.129, *p* = 0.024). ACE-R itself was not an independent correlate (*B* = 0.046, −0.019–0.110, *β* = 0.079, *p* = 0.161).

## 4 Discussion

This study is one of the first to examine the levels and factors contributing to caregiver distress related to NPS in the context of MBI (as defined in the ISTAART-AA criteria) in MCI-AD patients. In this context, we introduce the MBI-D, a brief informant-rated scale that specifically quantifies caregiver distress attributable to MBI domains in individuals with MCI-AD. In our study, three findings stand out. First, MBI-D total score showed a strong association with MBI-C total score, and each MBI-D domain score correlated robustly with its matching MBI-C domain score, supporting the intended symptom-distress coupling in prodromal AD. Second, in multivariable analysis, MBI-C total score and education could independently predict caregiver distress. When MBI-C total was replaced by the MBI-C domains, three domains independently correlated with greater distress: impulse dyscontrol, decreased motivation, and emotional dysregulation. Third, disease duration showed a positive association with distress at the bivariate level for MBI-D total and apathy domain, and trending positively in the multivariable models. These results suggest that longer exposure, particularly to apathy-related changes, may incrementally increase caregiver strain even before dementia onset.

Despite the well-known relationship between NPS and caregiver burden in dementia, literature evidence in pre-dementia populations is limited. In a cohort of patients with MCI and SCD, MBI was related to higher caregivers’ burden, evaluated by ZBI (Sheikh et al., 2018). In MCI, overall caregiver burden evaluated by CBI was lower compared to that of AD dementia, yet higher compared to that of cognitively normal controls (Ryan et al., 2012). The co-occurrence of NPS was linked to increased caregiver burden in MCI in this study (Ryan et al., 2012). A dose–response relationship was also observed, with increasing MBI symptoms severity correlated with greater caregiver burden (Ryan et al., 2012). In accordance, NPS frequency and severity in patients with MCI could predict the level of caregivers’ distress in another study, assessed by the NPI-D (Trivedi et al., 2013). A dose–response impact of NPS was also demonstrated on caregiving time in individuals in the pre-dementia or dementia stage (Okura and Langa, 2011), and behavioral disturbances contributed the most in caregiver burden among MCI patients in another study (Paradise et al., 2015). These findings agree with our results, since increasing MBI-C domain scores were significantly correlated with each of their corresponding MBI-D scores, and MBI-C total score was the main independent contributor to MBI-related caregiver distress. These findings highlight the importance of targeting the MBI symptom burden for preventing or managing caregiving distress in MCI-AD.

Caregiver distress was not uniformly distributed across MBI domains. When all domains were considered simultaneously (adjusting for age, education, sex, cognition, and disease duration), only impulse dyscontrol, decreased motivation, and emotional dysregulation showed independent associations with higher MBI-D total scores, suggesting that these MBI domains are the primary drivers of caregiver strain in MCI-AD. In this context, a recent study investigating caregiver burden evaluated by ZBI in individuals with SCD and MCI demonstrated that all MBI domains were individually associated with higher burden, with abnormal perception or thought content showing one of the highest burden levels, despite its lower frequency in the population (Sheikh et al., 2018). In this study, each MBI domain was modeled separately. In contrast, we entered all five MBI-C domain severities simultaneously to quantify their independent, mutually adjusted, effects on distress. Under this stricter criterion, impulse dyscontrol, decreased motivation and emotional dysregulation retained independent associations with higher distress, whereas social inappropriateness and abnormal perception or thought content did not. These different results can be explained by the assumption that psychotic symptoms such as visual hallucinations are not core features in MCI-AD, compared to MCI due to other neurodegenerative diseases, like Lewy body disease, in which they may often exist even in isolation (Donaghy et al., 2023). In line with our results, the only NPS with a significant discrepancy between dementia and MCI were hallucinations, with the dementia subgroup resulting in greater caregiver distress (Baiyewu et al., 2012). Taken together, while every MBI domain can relate to caregiver burden, impulsivity, apathy and affective dysregulation seem to independently drive distress in MCI-AD, thereby refining targets for domain-focused intervention in this subpopulation. Moreover, as the population frequency of MBI domains should inform public health planning, targeting the three most prevalent -and simultaneously most distressing-domains as indicated by our results, represents the probably most prudent and effective intervention strategy.

Supplementary correlation analyses showed that all MBI-D domains were significantly associated with overall MBI symptom burden, with affective dysregulation, decreased motivation, and impulse dyscontrol exhibiting the strongest associations. These findings are consistent with the primary multivariable analyses, in which the same three domains emerged as the main independent contributors to caregiver distress. Together, the convergent results across complementary analytical approaches strengthen the evidence that affective, motivational, and impulse control symptoms represent the core drivers of caregiver burden in MCI-AD.

Patient education years were positively associated with caregiver distress in the global MBI model, domain-level model, and ACE-R model. Although unexpected, several mechanisms could plausibly account for these results. Higher premorbid attainment may sharpen the discrepancy between prior functioning and current behavioral change, heightening caregiver appraisal of loss and role disruption. Patients with higher education, and often more complex daily roles, may sustain higher expectations for independence and adherence, so apathy or impulsivity can translate into greater conflict and monitoring burden. Furthermore, greater health literacy may increase recognition and/or reporting of MBI-related strain. Finally, residual confounding by caregiver characteristics, such as caregiver’s own education, employment or time constraints and socioeconomic opportunity costs may contribute. Given the modest effect size and our cross-sectional design, these findings should be interpreted cautiously. Nonetheless, they underscore the need to screen for and address distress irrespective of educational background, with expectation management and tailored psychoeducation for dyads in which premorbid achievement standards are high.

The absence of associations with global cognition (MMSE, ACE-R) in our study is consistent with the notion that behavioral symptoms and caregiver distress are not linear correlations of performance in cognitive tests in prodromal disease, highlighting the importance of systematically assessing NPS beyond cognition. In dementia, caregivers’ burden is affected by NPS to a greater extent compared to cognitive function (Torrisi et al., 2017). In pre-dementia populations, although caregiver burden was higher in MCI than in SCD, this difference appears largely driven by the presence of MBI: among individuals without MBI, caregiver burden does not differ significantly between MCI and SCD (Sheikh et al., 2018). These findings highlight the pivotal role of MBI in caregivers’ distress in MCI, suggesting also a possible “synergistic” impact of NPS and cognitive deficits on caregiving burden. As we did not include individuals with SCD in our study, future work should further explore this assumption.

Importantly, the absence of associations between MBI-D scores and measures of global cognition should also be interpreted in light of the instrument’s conceptual scope. The MBI-D was specifically designed to capture caregiver distress attributable to behavioral and NPS within the MBI framework, and it does not assess distress related to cognitive symptoms per se. Therefore, the lack of correlation with MMSE and ACE-R is consistent with the scale’s intended focus and should not be considered unexpected. While caregiver distress related to cognitive decline is undoubtedly clinically relevant, this construct falls outside the current scope of the MBI-D. Future work could extend the MBI framework by developing complementary instruments specifically designed to assess caregiver burden related to cognitive symptoms in prodromal disease, enabling a more comprehensive and integrated evaluation of caregiver impact across both behavioral and cognitive domains.

Concerning the different cognitive domains, MCI patients’ executive function has been associated with greater levels of caregiver burden evaluated by CBI (Ryan et al., 2012). Hence, greater executive dysfunction might also increase the levels of MBI-related caregiver distress in MCI. In our study, we only tested global cognition with MMSE and ACE-R, without performing detailed neuropsychological testing for cognitive domains. Also, our study participants involved patients with MCI-AD, whose primary cognitive deficits are amnestic and not dysexecutive. In addition, amnestic and non-amnestic MCI patients present with diverse patterns of NPS, which may affect the severity of MBI overall and domain-specific caregiver burden (Ryan et al., 2012). Since this research question was beyond the scope of our work, future studies should explore the mediating role of memory, executive function and other cognitive domains in MBI-related caregiver distress in MCI due to not only AD but also other underlying conditions, including vascular pathology or Lewy body disease.

In our study, longer disease duration was also associated with greater MBI total caregiver distress. However, although duration showed a positive trend in multivariable models, this association was not statistically significant. These differences suggest that the duration-distress relationship is largely conveyed through greater MBI symptom burden, such that, once overall or domain-specific MBI severity is accounted for, duration contributes little unique variance. This attenuation is unlikely to reflect multicollinearity (VIF ≈1.0–2.4). Nevertheless, given the cross-sectional design of our study, potential recall bias regarding the onset of symptoms should be considered, and longitudinal studies will provide more precise estimations about the role of disease duration in caregiver distress in MBI.

Consistent with prior work, our data highlight apathy as a pivotal driver of caregiver strain in prodromal AD. At the bivariate level, apathy-related distress correlated with disease duration, and in multivariable models, apathy retained an independent association with MBI-D. These findings agree with a recent longitudinal study showing that apathy -but not depression- at baseline could predict worsening of caregiver burden over time in MCI, measured by ZBI (Connors et al., 2023). Collectively, the evidence suggests that apathy imposes a sustained and accumulating load on caregivers: it is common early, associates with longer symptom exposure, and exerts a unique, domain-specific effect on distress even after accounting for co-occurring behavioral changes. Clinically, this supports early, targeted interventions for apathy (e.g., structured activity scheduling, caregiver skills training, environmental cueing), which may be particularly impactful for preventing escalation of burden as the disease progresses.

In dementia although apathy is highly prevalent, caregiver distress is more strongly driven by disruptive or unsafe behaviors, such as irritability, agitation, sleep–wake disturbances, and psychotic symptoms (Terum et al., 2017; Allegri et al., 2006; Tun et al., 2008). These behaviors impose immediate and high-intensity demands, including continuous supervision, sleep disruption, safety concerns, and repeated negotiation around essential care, thereby generating disproportionate caregiver strain. By contrast, apathy, while functionally impairing, is often more predictable and may be partially accommodated through prompting or routine adjustments, resulting in comparatively lower perceived burden in later disease stages. In MCI-AD, however, apathy may be particularly distressing, as caregivers often interpret it as a motivational or volitional change rather than disease-related incapacity. Higher expectations regarding autonomy, adherence, and engagement in compensatory strategies, combined with limited caregiver preparedness in early disease stages, may amplify frustration and distress. Together, these factors may explain why apathy emerges as a prominent driver of caregiver distress in prodromal AD, while being relatively overshadowed by more disruptive symptoms in dementia.

Prior work has shown that anosoagnosia, as well as apathy and agitation, are associated with reduced caregiver quality of life in dementia (Conde-Sala et al., 2016) and MCI populations (Mak et al., 2015), likely reflecting the shared disruption of frontal-subcortical networks (Le Heron et al., 2018). Given the pathophysiological overlap between apathy and executive dysfunction, it is plausible that early executive impairment may contribute to apathy-related caregiver distress in prodromal disease. However, as anosognosia and executive functioning were not directly assessed in the present study, these associations remain speculative. Future studies incorporating targeted measures of insight and executive function are needed to clarify their contribution to MBI-related caregiver distress.

From a construct perspective, as intended for a brief, five-item instrument for distinct MBI domains, the MBI-D showed moderate internal consistency (items are not meant to be redundant). In contrast, the NPI-D comprises many more items aligned with the full NPI domain set; accordingly, a high Cronbach’s alpha for the NPI-D is typically reported (Davidsdottir et al., 2012), which is expected because alpha increases with both the number of items and the average inter-item correlation (McCrae et al., 2011). Importantly, for instruments such as the MBI-D, which are designed to capture related but conceptually distinct domains, very high internal consistency is neither theoretically expected nor psychometrically required. In this context, moderate alpha values indicate that items are sufficiently related to justify a total score, while still preserving domain specificity and avoiding redundancy. Accordingly, the interpretation of internal consistency for the MBI-D should emphasize construct coverage and clinical interpretability rather than unidimensionality.

In our study, we could not assess test–retest or inter-rater reliability because repeated assessments and multiple raters were not obtained. Nor could we establish criterion validity against an MBI-specific distress measure, as none exists. In addition, no quality-of-life or mood measures were included, which would have allowed a more comprehensive evaluation of convergent validity with psychosocial caregiver outcomes. Nevertheless, content validity was supported by expert review, and convergent validity was evidenced by the strong coupling between MBI-C (symptom severity) and MBI-D (domain-matched distress) at both total and domain levels. Future work should evaluate temporal stability (test–retest), inter-rater agreement, minimal clinically important difference, and convergence with established caregiver-reported outcomes, including quality of life, depressive symptoms, and emotional well-being. Furthermore, triangulation with established but non-MBI-specific caregiver burden scales, such as NPI-D, ZBI or CBI could further benchmark convergent and discriminant validity, while acknowledging differences in construct focus.

Pairing the MBI-C with the new MBI-D is informative and practical for the clinical settings, since in a single administration clinicians obtain both MBI symptom severity (MBI-C) and domain-specific caregiver distress (MBI-D). The MBI-D was built by integrating two validated frameworks, the MBI-C and the NPI-, thereby ensuring the alignment with research in NPS in the spectrum of cognitive impairment. Unlike tools commonly used in prodromal cohorts, which are either dementia-oriented or nonspecific to NPS, the combined MBI-C + MBI-D approach is MBI-specific, time-efficient and more appropriate for early-stage, functionally independent patients.

Higher caregiver burden in MCI has been associated with lower levels of life satisfaction, less social support and an increased need for support services (Ryan et al., 2012). These findings combined with our results suggest that providing family members and caregivers of MCI-AD patients with the available resources, support services and psychoeducation on managing behavioral and neuropsychiatric difficulties might mitigate their burden early during the disease course.

Importantly, literature evidence indicates a bidirectional linkage between NPS and caregiver burden (Isik et al., 2019). Agitation, irritability, disinhibition and aggression are major drivers of caregiver burden and quality of life. In turn, higher caregiver burden and strained caregiver-patient interactions can worsen the frequency and/or severity of NPS, creating a self-reinforcing cycle of distress (Isik et al., 2019). Accordingly, active management should target both sides: early identification and treatment of NPS and mitigation of caregiver stress, since improvement in one domain can benefit the other.

The World Health Organization’s global action plan for dementia emphasizes the importance of supporting caregivers to preserve well-being. In our pre-dementia cohort, longer disease duration was associated with greater caregiver distress, particularly distress related to decreased motivation, an MBI domain that often emerges early. These findings support the need for earlier intervention, before cumulative exposure amplifies strain. Paired screening with the MBI-C and MBI-D enables early identification of both NPS and their caregiver impact, facilitating timely, non-pharmacological interventions within routine memory-clinic pathways (e.g., psychoeducation, apathy-focused skills training, environmental modifications, and linkage to support services). Although causal inference is limited by the cross-sectional design, the observed duration-distress association provides a strong rationale for acting early rather than waiting for later disease stages.

The combined use of the MBI-C and MBI-D represents a scalable and implementation-ready innovation for dementia services. As a brief, two-page assessment package that can be integrated into routine intake or follow-up encounters, it provides immediate, domain-level indicators of caregiver impact that can guide tailored, non-pharmacological care strategies. By identifying the specific behavioral domains that independently drive caregiver distress, the model supports more efficient triage and resource allocation; for example, apathy-related distress can prompt referral to activation-based occupational therapy, while distress linked to impulse dyscontrol may highlight the need for caregiver skills training or environmental modification strategies. Importantly, this approach is feasible in settings without biomarker access, making it particularly relevant for memory clinics, geriatric services, and telemedicine pathways in resource-limited regions. Moreover, the MBI-D offers a practical outcome metric -reduction in caregiver distress-that can be used for service evaluation, commissioning, and value-based care planning. Thus, integrating the MBI-D into clinical workflows aligns with contemporary priorities for earlier, equitable, and caregiver-centered dementia prevention and care.

In our study, we only used subjective measures of caregiver burden, involving the perceived distress evaluated by MBI-D. It would be useful for future studies to assess objective burden, such as time and/or intensity of care tasks, as well as the use of caregivers’ resources (Cao and Yang, 2020). This approach would help holistically interpret MBI distress, thereby contributing to more efficient management planning.

In this study, we applied an MBI-C cutoff score of 9.5, derived from our previous work in the same Greek cohort, where it demonstrated optimal discriminative ability for identifying MBI in individuals with MCI-AD (Angelopoulou et al., 2025). The slightly higher cutoff compared to that previously proposed in broader MCI populations likely reflects the greater severity and persistence of NPS specifically in MCI-AD, which may translate into more clinically meaningful behavioral change. Importantly, this threshold aligns with our core findings, as individuals exceeding the MBI-C cutoff of 9.5 exhibited substantially higher levels of caregiver distress, and MBI-C total score emerged as the main independent predictor of MBI-related caregiver distress in multivariable analyses. Taken together, these findings support the need for a population- and disease-specific calibration of MBI-C thresholds to ensure optimal clinical relevance, and underscore the value of pairing symptom severity with domain-specific caregiver distress when studying prodromal AD. Our study has certain limitations. Participants were recruited from a single memory clinic in Greece, which may limit generalizability. We did not collect caregiver-level demographic or contextual determinants, such as age, sex, education, relationship to patient, cohabitation, caregiving hours, or caregiver mood, which could influence the experience and reporting of burden. Prior work has shown that psychiatric vulnerability, personality characteristics and coping capacity may affect caregivers’ distress and its evolution (Torrisi et al., 2017). Furthermore, emotional expression, family dynamics, social norms and support systems may vary across sociocultural contexts, thereby affecting NPS interpretation, coping strategies and caregiver burden. While internal consistency was moderate for a brief heterogeneous scale, future studies should validate the MBI-D across diverse caregiving populations and cultural settings, and examine its convergent, discriminant, and predictive validity in relation to caregiver outcomes, such as depression and quality of life, as well as patient trajectories, including functional decline, institutionalization, and NPS progression.

Our MCI-AD diagnoses followed the 2011 NIA-AA clinical criteria, rather than the 2024 NIA-AA research framework that integrates biomarkers (Jack et al., 2024). Although biomarkers [cerebrospinal fluid (CSF), amyloid/tau positron emission tomography (PET), blood-based assays] can improve diagnostic precision, their limited availability especially in underserved areas underscores the ongoing importance of clinical diagnosis combined with the use of the MBI-C and MBI-D in routine care.

Nonetheless, our study has several strengths, including a well-characterized MCI-AD cohort, standardized neuropsychological assessment, content validation by experts, and the use of a multivariable model to examine which patients’ factors independently affect MBI caregiver distress. We did not use general measures of caregiver burden, but a newly developed scale to specifically capture MBI-related caregiver distress. In addition, most studies investigating caregiver burden in MCI are based in tertiary academic centers (Sheikh et al., 2018), limiting the generalizability of their results to primary care-community settings.

## 5 Conclusion

In this well-characterized MCI-AD cohort, we introduce and validate the MBI-D, a brief, domain-matched measure of caregiver distress aligned to the ISTAART-AA MBI framework. The scale showed moderate internal consistency appropriate for a five-item, non-redundant instrument, and demonstrated robust convergent validity with the MBI-C at both total and domain levels. Across models, MBI burden -particularly impulse dyscontrol, apathy, and emotional dysregulation- emerged as the primary independent drivers of caregiver distress, whereas global cognition measures were not independently associated. Disease duration correlated with overall and apathy-related distress in bivariate analyses but contributed limited unique variance after accounting for MBI severity, suggesting that cumulative exposure exerts its effect largely through greater symptom burden. A small but consistent positive association of education with distress warrants further exploration in future work.

Clinically, pairing MBI-C + MBI-D enables time-efficient, domain-specific profiling of symptoms and caregiver impact in routine practice, supporting early, targeted non-pharmacological strategies. Next steps should include assessment of test–retest and inter-rater reliability, minimal clinically important difference, as well as incorporation of objective burden indices and caregiver-level determinants. If validated longitudinally and across settings, the MBI-D can help standardize early identification of behavior-linked distress and inform personalized intervention pathways in prodromal AD.

## Data Availability

The original contributions presented in the study are included in the article/Supplementary material, further inquiries can be directed to the corresponding author.

## References

[ref1] Allegri R. F. Sarasola D. Serrano C. M. Taragano F. E. Arizaga R. L. Butman J. . (2006). Neuropsychiatric symptoms as a predictor of caregiver burden in Alzheimer’s disease. Neuropsychiatr. Dis. Treat. 2, 105–110. 19412452 PMC2671738

[ref2] Angelopoulou E. Stanitsa E. Hatzopoulou M. Despoti A. Tsinia N. Kamtsadeli V. . (2025). The Greek version of the mild behavioral impairment checklist (MBI-C): psychometric properties in mild cognitive impairment due to Alzheimer’s disease. Brain Sci. 15:462. doi: 10.3390/brainsci15050462, 40426633 PMC12110333

[ref3] Baiyewu O. Unverzagt F. W. Ogunniyi A. Smith-Gamble V. Gureje O. Lane K. A. . (2012). Behavioral symptoms in community-dwelling elderly Nigerians with dementia, mild cognitive impairment, and normal cognition. Int. J. Geriatr. Psychiatry 27, 931–939. doi: 10.1002/gps.2804, 22383107 PMC3418445

[ref4] Basu I. Mukhopadhyay S. (2022). Neuropsychiatric symptoms of dementia and caregivers’ burden: a study among Indian caregivers. Dement. Neuropsychol. 16, 332–340. doi: 10.1590/1980-5764-DN-2022-0017, 36619839 PMC9762380

[ref5] Cao Y. Yang F. (2020). Objective and subjective dementia caregiving burden: the moderating role of immanent justice reasoning and social support. Int. J. Environ. Res. Public Health 17:455. doi: 10.3390/ijerph17020455, 31936738 PMC7014207

[ref6] Chen Y. Dang M. Zhang Z. (2021). Brain mechanisms underlying neuropsychiatric symptoms in Alzheimer’s disease: a systematic review of symptom-general and -specific lesion patterns. Mol. Neurodegener. 16:38. doi: 10.1186/s13024-021-00456-1, 34099005 PMC8186099

[ref7] Conde-Sala J. L. Turró-Garriga O. Piñán-Hernández S. Portellano-Ortiz C. Viñas-Diez V. Gascón-Bayarri J. . (2016). Effects of anosognosia and neuropsychiatric symptoms on the quality of life of patients with Alzheimer’s disease: a 24-month follow-up study. Int. J. Geriatr. Psychiatry 31, 109–119. doi: 10.1002/gps.4298, 25963296

[ref8] Connors M. H. Teixeira-Pinto A. Ames D. Woodward M. Brodaty H. (2023). Apathy and depression in mild cognitive impairment: distinct longitudinal trajectories and clinical outcomes. Int. Psychogeriatr. 35, 633–642. doi: 10.1017/S1041610222001089, 36715000

[ref9] Davidsdottir S. R. Snaedal J. Karlsdottir G. Atladottir I. Hannesdottir K. (2012). Validation of the Icelandic version of the Neuropsychiatric Inventory with Caregiver Distress (NPI-D). Nord. J. Psychiatry 66, 26–32. doi: 10.3109/08039488.2011.593100, 21770826

[ref10] Donaghy P. C. Carrarini C. Ferreira D. Habich A. Aarsland D. Babiloni C. . (2023). Research diagnostic criteria for mild cognitive impairment with Lewy bodies: a systematic review and meta-analysis. Alzheimers Dement. 19, 3186–3202. doi: 10.1002/alz.13105, 37096339 PMC10695683

[ref11] Huang S.-S. (2022). Depression among caregivers of patients with dementia: associative factors and management approaches. World J. Psychiatry 12, 59–76. doi: 10.5498/wjp.v12.i1.59, 35111579 PMC8783169

[ref12] Isik A. T. Soysal P. Solmi M. Veronese N. (2019). Bidirectional relationship between caregiver burden and neuropsychiatric symptoms in patients with Alzheimer’s disease: a narrative review. Int. J. Geriatr. Psychiatry 34, 1326–1334. doi: 10.1002/gps.4965, 30198597

[ref13] Ismail Z. Agüera-Ortiz L. Brodaty H. Cieslak A. Cummings J. Fischer C. E. . (2017). The mild behavioral impairment checklist (MBI-C): a rating scale for neuropsychiatric symptoms in pre-dementia populations. J. Alzheimers Dis. 56, 929–938. doi: 10.3233/JAD-160979, 28059789 PMC5652315

[ref14] Jack C. R. Andrews J. S. Beach T. G. Buracchio T. Dunn B. Graf A. . (2024). Revised criteria for diagnosis and staging of Alzheimer’s disease: Alzheimer’s association workgroup. Alzheimers Dement. 20, 5143–5169. doi: 10.1002/alz.13859, 38934362 PMC11350039

[ref15] Jack C. R. Bennett D. A. Blennow K. Carrillo M. C. Dunn B. Haeberlein S. B. . (2018). NIA-AA research framework: toward a biological definition of Alzheimer’s disease. Alzheimers Dement. 14, 535–562. doi: 10.1016/j.jalz.2018.02.018, 29653606 PMC5958625

[ref16] Kaufer D. I. Cummings J. L. Christine D. Bray T. Castellon S. Masterman D. . (1998). Assessing the impact of neuropsychiatric symptoms in Alzheimer’s disease: the neuropsychiatric inventory caregiver distress scale. J. Am. Geriatr. Soc. 46, 210–215. doi: 10.1111/j.1532-5415.1998.tb02542.x, 9475452

[ref17] Le Heron C. Apps M. A. J. Husain M. (2018). The anatomy of apathy: a neurocognitive framework for amotivated behaviour. Neuropsychologia 118, 54–67. doi: 10.1016/j.neuropsychologia.2017.07.003, 28689673 PMC6200857

[ref18] Lyketsos C. G. Lopez O. Jones B. Fitzpatrick A. L. Breitner J. DeKosky S. (2002). Prevalence of neuropsychiatric symptoms in dementia and mild cognitive impairment: results from the cardiovascular health study. JAMA 288, 1475–1483. doi: 10.1001/jama.288.12.1475, 12243634

[ref19] Mak E. Chin R. Ng L. T. Yeo D. Hameed S. (2015). Clinical associations of anosognosia in mild cognitive impairment and Alzheimer’s disease. Int. J. Geriatr. Psychiatry 30, 1207–1214. doi: 10.1002/gps.4275 25754519

[ref20] Mank A. van Maurik I. S. Rijnhart J. J. M. Rhodius-Meester H. F. M. Visser L. N. C. Lemstra A. W. . (2023). Determinants of informal care time, distress, depression, and quality of life in care partners along the trajectory of Alzheimer’s disease. Alzheimers Dement. Amst. Neth. 15:e12418. doi: 10.1002/dad2.12418, 37114014 PMC10126754

[ref21] McCrae R. R. Kurtz J. E. Yamagata S. Terracciano A. (2011). Internal consistency, retest reliability, and their implications for personality scale validity. Personal. Soc. Psychol. Rev. 15, 28–50. doi: 10.1177/1088868310366253, 20435807 PMC2927808

[ref22] Okura T. Langa K. M. (2011). Caregiver burden and neuropsychiatric symptoms in older adults with cognitive impairment: the aging, demographics, and memory study (ADAMS). Alzheimer Dis. Assoc. Disord. 25, 116–121. doi: 10.1097/WAD.0b013e318203f208, 21192239 PMC3100441

[ref23] Paradise M. McCade D. Hickie I. B. Diamond K. Lewis S. J. G. Naismith S. L. (2015). Caregiver burden in mild cognitive impairment. Aging Ment. Health 19, 72–78. doi: 10.1080/13607863.2014.915922 24866046

[ref24] Politis A. M. Mayer L. S. Passa M. Maillis A. Lyketsos C. G. (2004). Validity and reliability of the newly translated Hellenic Neuropsychiatric Inventory (H-NPI) applied to Greek outpatients with Alzheimer’s disease: a study of disturbing behaviors among referrals to a memory clinic. Int. J. Geriatr. Psychiatry 19, 203–208. doi: 10.1002/gps.1045, 15027034

[ref25] Rosenberg P. B. Mielke M. M. Appleby B. S. Oh E. S. Geda Y. E. Lyketsos C. G. (2013). The association of neuropsychiatric symptoms in MCI with incident dementia and Alzheimer disease. Am. J. Geriatr. Psychiatry 21, 685–695. doi: 10.1016/j.jagp.2013.01.006, 23567400 PMC3428504

[ref26] Ryan K. A. Weldon A. Persad C. Heidebrink J. L. Barbas N. Giordani B. (2012). Neuropsychiatric symptoms and executive functioning in patients with mild cognitive impairment: relationship to caregiver burden. Dement. Geriatr. Cogn. Disord. 34, 206–215. doi: 10.1159/000339955, 23128102 PMC3698846

[ref27] Saari T. Koivisto A. Hintsa T. Hänninen T. Hallikainen I. (2022). Psychometric properties of the neuropsychiatric inventory: a review. J. Alzheimers Dis. 86, 1485–1499. doi: 10.3233/JAD-200739, 32925068 PMC9108559

[ref28] Sheikh F. Ismail Z. Mortby M. E. Barber P. Cieslak A. Fischer K. . (2018). Prevalence of mild behavioral impairment in mild cognitive impairment and subjective cognitive decline, and its association with caregiver burden. Int. Psychogeriatr. 30, 233–244. doi: 10.1017/S104161021700151X, 28879833

[ref29] Steinberg M. Shao H. Zandi P. Lyketsos C. G. Welsh-Bohmer K. A. Norton M. C. . (2008). Point and 5-year period prevalence of neuropsychiatric symptoms in dementia: the Cache County Study. Int. J. Geriatr. Psychiatry 23, 170–177. doi: 10.1002/gps.1858, 17607801 PMC2932652

[ref30] Terum T. M. Andersen J. R. Rongve A. Aarsland D. Svendsboe E. J. Testad I. (2017). The relationship of specific items on the neuropsychiatric inventory to caregiver burden in dementia: a systematic review. Int. J. Geriatr. Psychiatry 32, 703–717. doi: 10.1002/gps.4704, 28317166

[ref31] Torrisi M. De Cola M. C. Marra A. De Luca R. Bramanti P. Calabrò R. S. (2017). Neuropsychiatric symptoms in dementia may predict caregiver burden: a Sicilian exploratory study. Psychogeriatrics 17, 103–107. doi: 10.1111/psyg.12197, 27411501

[ref32] Trivedi S. C. Subramanyam A. A. Pinto C. Gambhire D. D. (2013). Neuropsychiatric symptoms in mild cognitive impairment: an analysis and its impact on caregiving. Indian J. Psychiatry 55, 154–160. doi: 10.4103/0019-5545.111454, 23825850 PMC3696239

[ref33] Tu J. Y. Jin G. Chen J.-H. Chen Y.-C. (2022). Caregiver burden and dementia: a systematic review of self-report instruments. J. Alzheimers Dis. 86, 1527–1543. doi: 10.3233/JAD-215082, 35253744

[ref34] Tun S.-M. Murman D. L. Colenda C. C. (2008). Concurrent validity of neuropsychiatric subgroups on caregiver burden in Alzheimer disease patients. Am. J. Geriatr. Psychiatry 16, 594–602. doi: 10.1097/JGP.0b013e318173f5fc, 18591579

[ref35] Zhang S. Ying X. Fang S. Wang W. Zhu X. Dong Y. . (2022). The influence path of caregivers’ positive aspects, expressed emotion and coping style on behavioral and psychological symptoms of dementia. Geriatr. Nurs. 44, 143–150. doi: 10.1016/j.gerinurse.2022.01.013, 35158171

