# Supplementary material for: From mild behavioral impairment-checklist (MBI-C) to MBI-distress (MBI-D): a paired assessment and clinical correlates of domain-specific caregiver distress in MCI due to AD
Source: Front Dement. 2026 Feb 24;5:1736570. doi: 10.3389/frdem.2026.1736570 (PMC12974401; doi:10.3389/frdem.2026.1736570)
Supplement: Supplementary file 1 [file Table_1.pdf]

The developed Greek MBI-D version, which accompanies MBI-C, and a translated English version are depicted in Tables A1, A2.

Table A1. MBI-D questions in Greek

| **Ερωτηματολόγιο Ήπιας Συμπεριφορικής Διαταραχής—Mild Behavioral Impairment—Checklist (MBI-C) (ελληνική έκδοση, μετάφραση: Ι. Παπατριανταφύλλου, MD, PhD)** | | | | | | |
|---|---|---|---|---|---|---|
| **ΟΝΟΜΑ:** | | | | | | |
| **ΗΜΕΡΟΜΗΝΙΑ:** | | | | | | |
| **Ερευνήσατε αν τους τελευταίους 6 μήνες υπάρχουν τα παρακάτω συμπτώματα και βαθμολογήσατε την σοβαρότητά τους** | **ΣΟΒΑΡΟΤΗΤΑ** | | | | | |
| **0 δεν υπάρχει, 1 ήπια, 2 μέτρια, 3 σοβαρή (σε αμφιβολία την σοβαρότερη βαθμολογία)** | | | | | | |
| **AFF Αυτό το πεδίο περιγράφει τη διάθεση και τα συμπτώματα άγχους** | *όχι* | | *ναι* | | | |
| 1. Το άτομο έχει αναπτύξει θλίψη ή φαίνεται έχει κακή διάθεση; Έχει επεισόδια που κλαίει/ξεσπά σε κλάματα; | 0 | 1 | 2 | 3 | | |
| 2. Το άτομο δυσκολεύεται/είναι λιγότερο ικανό να αντλήσει ευχαρίστηση; | 0 | 1 | 2 | 3 | | |
| 3. Το άτομο είναι αποθαρρημένο για το μέλλον του ή νιώθει αποτυχημένος; | 0 | 1 | 2 | 3 | | |
| 4. Το άτομο βλέπει τον εαυτό του ως βάρος για την οικογένειά του; | 0 | 1 | 2 | 3 | | |
| 5. Το άτομο είναι περισσότερο αγχωμένο ή ανήσυχο για πράγματα που αποτελούν ρουτίνα (π.χ. εκδηλώσεις, επισκέψεις); | 0 | 1 | 2 | 3 | | |
| 6. Το άτομο νιώθει μεγάλη ένταση, αδυνατεί να χαλαρώσει, παρουσιάζει αστάθεια/τρόμο ή έχει συμπτώματα πανικού; | 0 | 1 | 2 | 3 | | |
| **MBI-D Εάν υπάρχουν, πόσο συναισθηματικά επιβαρυντικά ήταν για εσάς τα συμπτώματα συναισθηματικών μεταβολών;** | 0 Καθόλου | 1 Ελάχιστα | 2 Ήπια | 3 Μέτρια | 4 Σοβαρά | 5 Πολύ Σοβαρά |

| | | | | | | |
|---|---|---|---|---|---|---|
| | | | | | | |
| **MOT Αυτό το πεδίο περιγράφει το ενδιαφέρον, την παρακίνηση και τη δράση/ενέργεια** | *όχι* | | | *ναι* | | |
| 1. Το άτομο έχει χάσει το ενδιαφέρον για τους φίλους, την οικογένεια ή τις δραστηριότητες του σπιτιού; | 0 | 1 | 2 | 3 | | |
| 2. Το άτομο έχει χάσει την περιέργεια του για θέματα που συνήθως τραβούσαν το ενδιαφέρον του/της; | 0 | 1 | 2 | 3 | | |
| 3. Το άτομο έχει γίνει λιγότερο αυθόρμητο ή ενεργό/ δραστήριο- για παράδειγμα είναι λιγότερο πιθανό να ξεκινήσει ή να διατηρήσει μία συζήτηση; | 0 | 1 | 2 | 3 | | |
| 4. Το άτομο έχει χάσει το κίνητρό για τις υποχρεώσεις που είχε μέχρι τώρα και για τα ενδιαφέροντά του; | 0 | 1 | 2 | 3 | | |
| 5. Το άτομο είναι λιγότερο στοργικό και/ή χωρίς συναισθηματική ανταπόκριση σε σχέση με παλαιότερα; | 0 | 1 | 2 | 3 | | |
| 6. Πλέον δεν νοιάζεται για τίποτα; | 0 | 1 | 2 | 3 | | |
| **MBI-D Εάν υπάρχουν, πόσο συναισθηματικά επιβαρυντικά ήταν για εσάς τα συμπτώματα μειωμένου κινήτρου;** | 0<br>Καθόλου | 1<br>Ελάχιστα | 2<br>Ήπια | 3<br>Μέτρια | 4<br>Σοβαρά | 5<br>Πολύ Σοβαρά |
| | | | | | | |
| **IMP Αυτό το πεδίο περιγράφει την ικανότητα καθυστέρησης στην λήψη ικανοποίησης, τον έλεγχο της συμπεριφοράς, την παρόρμηση, την πρόσληψη τροφής και/ή αλλαγές στην επιβράβευση** | *όχι* | | | *ναι* | | |
| | | | | | | |
| 1. Το άτομο έχει γίνει ανήσυχο, επιθετικό, ευερέθιστο ή κυκλοθυμικό; | 0 | 1 | 2 | 3 | | |
| 2.Έχει γίνει αναίτια ή περίεργα/ ιδιαίτερα εριστικό; | 0 | 1 | 2 | 3 | | |
| 3.Το άτομο έχει γίνει περισσότερο παρορμητικό, φαίνεται ως εάν να λειτουργεί χωρίς να υπολογίζει τίποτα; | 0 | 1 | 2 | 3 | | |
| 4.Το άτομο παρουσιάζει σεξουαλικές απαγορευμένες ή αδιάκριτες συμπεριφορές, όπως το να ακουμπάει (τον εαυτό του ή άλλους), να αγκαλιάζει, θωπεύει κ.α., με τρόπο που δεν είναι στο | 0 | 1 | 2 | 3 | | |

| | | | | |
|---|---|---|---|---|
| χαρακτήρα του και μπορεί να προκαλέσει παράπτωμα; | | | | |
| 5.Το άτομο απογοητεύεται πιο εύκολα ή είναι πιο ανυπόμονο; Έχει δυσκολία να αντιμετωπίσει τις καθυστερήσεις ή να περιμένει σε εκδηλώσεις για τη σειρά του; | 0 | 1 | 2 | 3 |
| 6.Το άτομο παρουσιάζει μία καινοφανή απερισκεψία ή έλλειψη κρίσης όταν οδηγεί (π.χ. αναπτύσσει ταχύτητα, κάνει απρόβλεπτους ελιγμούς, απότομες αλλαγές στη λωρίδα κ.α.) | 0 | 1 | 2 | 3 |
| 7.Το άτομο έχει γίνει περισσότερο ισχυρογνώμων και άκαμπτο, π.χ. επιμένει πολύ στον δικό του τρόπο, δεν είναι πρόθυμο δεν είναι ικανό να δει ή να ακούσει άλλες οπτικές/απόψεις; | 0 | 1 | 2 | 3 |
| 8. Το άτομο συσσωρεύει/συγκεντρώνει αντικείμενα, κάτι που δεν έκανε στο παρελθόν; | 0 | 1 | 2 | 3 |
| 9.Το άτομο έχει αναπτύξει απλές επαναληπτικές συμπεριφορές ή ψυχαναγκασμούς; | 0 | 1 | 2 | 3 |
| 10.Το άτομο έχει πρόσφατα αναπτύξει δυσκολία να ελέγξει το κάπνισμά του, το αλκοόλ, τη λήψη ναρκωτικών ουσιών, τον τζόγο, ή ξεκίνησε την κλοπή από καταστήματα; | 0 | 1 | 2 | 3 |
| 11.Υπάρχει αλλαγή στις διατροφικές συνήθειες του ατόμου (π.χ. υπερφαγία, γεμίζει το στόμα του με φαγητό, επιμένει στο να τρώει μόνο συγκεκριμένα φαγητά ή να τρώει φαγητά με συγκεκριμένη σειρά); | 0 | 1 | 2 | 3 |
| 12.Το άτομο δεν βρίσκει πλέον το φαγητό νόστιμο ή ως κάτι που το ευχαριστεί; Τρώει λιγότερο; | 0 | 1 | 2 | 3 |

| | | | | | | |
|---|---|---|---|---|---|---|
| **MBI-D Εάν υπάρχουν, πόσο συναισθηματικά επιβαρυντικά ήταν για εσάς τα συμπτώματα παρορμητικής συμπεριφοράς;** | 0<br>Καθόλου | 1<br>Ελάχιστα | 2<br>Ήπια | 3<br>Μέτρια | 4<br>Σοβαρά | 5<br>Πολύ Σοβαρά |
| | | | | | | |
| **SOCCOG Αυτό το πεδίο περιγράφει τη συμμόρφωση στις κοινωνικές νόρμες και** | *όχι* | | | *ναι* | | |

| | | | | | | |
|---|---|---|---|---|---|---|
| **κοινωνική προσαρμογή, διακριτικότητα και ενσυναίσθηση** | | | | | | |
| 1.Το άτομο ανησυχεί λιγότερο για την επίδραση των λέξεων ή των πράξεων του στους άλλους; Έχει γίνει απαθής στα συναισθήματα των άλλων; | 0 | 1 | 2 | 3 | | |
| 2.Το άτομο έχει αρχίσει να μιλάει πιο ανοιχτά για πολύ προσωπικά ή ιδιωτικά του ζητήματα/θέματα που συνήθως δεν συζητούσε δημόσια; | 0 | 1 | 2 | 3 | | |
| 3.Το άτομο μιλά με αγενή ή ακατέργαστο τρόπο ή κάνει άσεμνα σεξουαλικού περιεχομένου σχόλια που δεν θα τα ανέφερε στο παρελθόν; | 0 | 1 | 2 | 3 | | |
| 4.Το άτομο φαίνεται να μη συμμορφώνεται στους κοινωνικούς κανόνες/ γνώμη κάτι που παλαιότερα ακολουθούσε στον δημόσιο ή ιδιωτικό χώρο; | 0 | 1 | 2 | 3 | | |
| 5.Το άτομο μιλάει σε ξένους ως εάν να του είναι γνωστοί, ή παρεμβαίνει στις δραστηριότητές τους; | 0 | 1 | 2 | 3 | | |
| **MBI-D Εάν υπάρχουν, πόσο συναισθηματικά επιβαρυντικά ήταν για εσάς τα συμπτώματα κοινωνικά ανάρμοστης συμπεριφοράς;** | 0 Καθόλου | 1 Ελάχιστα | 2 Ήπια | 3 Μέτρια | 4 Σοβαρά | 5 Πολύ Σοβαρά |
| | | | | | | |
| **PSY Αυτό το πεδίο περιγράφει βαθιά ριζωμένες πεποιθήσεις και αισθητηριακές εμπειρίες** | *όχι* | | | *ναι* | | |
| 1. Το άτομο αναφέρει ότι ακούει φωνές ή μιλά σε φανταστικούς ανθρώπους ή «πνεύματα»; | 0 | 1 | 2 | 3 | | |
| 2. Το άτομο αναφέρει ή παραπονιέται για/ή ενεργεί σαν να βλέπει πράγματα (π.χ. ανθρώπους, ζώα ή έντομα) που δεν βρίσκονται στο χώρο π.χ. που είναι φανταστικά/μη υπαρκτά για τους άλλους; | 0 | 1 | 2 | 3 | | |
| 3. Το άτομο έχει αναπτύξει πεποιθήσεις ότι βρίσκεται σε κίνδυνο, ή ότι οι άλλοι σχεδιάζουν να του/της κάνουν κακό ή να κλέψουν τα αντικείμενά τους; | 0 | 1 | 2 | 3 | | |
| Το άτομο έχει γίνει καχύποπτο για τις προθέσεις | 0 | 1 | 2 | 3 | | |

| | | | | | | |
|---|---|---|---|---|---|---|
| και τα κίνητρα των άλλων ανθρώπων; | | | | | | |
| 5. Το άτομο έχει μη ρεαλιστικές πεποιθήσεις για τις δυνάμεις του, την υγεία του ή τις ικανότητες του; | 0 | 1 | 2 | 3 | | |
| **MBI-D Εάν υπάρχουν, πόσο συναισθηματικά επιβαρυντικά ήταν για εσάς τα συμπτώματα παράδοξων σκέψεων ή αντιλήψεων;** | 0<br>Καθόλου | 1<br>Ελάχιστα | 2<br>Ήπια | 3<br>Μέτρια | 4<br>Σοβαρά | 5<br>Πολύ Σοβαρά |
| | | | | | | |
| **ΣΥΝΟΛΟ** | | | | | | |
