# Supplementary material for: From mild behavioral impairment-checklist (MBI-C) to MBI-distress (MBI-D): a paired assessment and clinical correlates of domain-specific caregiver distress in MCI due to AD
Source: Front Dement. 2026 Feb 24;5:1736570. doi: 10.3389/frdem.2026.1736570 (PMC12974401; doi:10.3389/frdem.2026.1736570)
Supplement: Supplementary file 2 [file Table_2.pdf]

Table A2. MBI-D questions in English

**Please rate severity: 0 = no distress; 1 = minimally distressing, 2 = mildly distressing, 3 = moderately distressing, 4 = severely distressing, 5 = very severely or extremely distressing**						
**MBI-D 1. If present, how emotionally distressing have the symptoms of emotional changes been for you?**	0<br>no distress	1<br>minimally distressing	2<br>mildly distressing	3<br>moderately distressing	4<br>severely distressing	5<br>very severely or extremely distressing
**MBI-D 2. If present, how emotionally distressing have the symptoms of reduced motivation been for you?**	0<br>no distress	1<br>minimally distressing	2<br>mildly distressing	3<br>moderately distressing	4<br>severely distressing	5<br>very severely or extremely distressing
**MBI-D 3. If present, how emotionally distressing have the impulsive behavioral symptoms been for you?**	0<br>no distress	1<br>minimally distressing	2<br>mildly distressing	3<br>moderately distressing	4<br>severely distressing	5<br>very severely or extremely distressing
**MBI-D 4. If present, how emotionally distressing have the impulsive behavioral symptoms been for you?**	0<br>no distress	1<br>minimally distressing	2<br>mildly distressing	3<br>moderately distressing	4<br>severely distressing	5<br>very severely or extremely distressing
**MBI-D 5. If present, how emotionally distressing have the symptoms of abnormal thoughts or perceptions been for you?**	0<br>no distress	1<br>minimally distressing	2<br>mildly distressing	3<br>moderately distressing	4<br>severely distressing	5<br>very severely or extremely distressing
**TOTAL:**						
